# Amniotic Fluid INSL3 Measured During the Critical Time Window in Human Pregnancy Relates to Cryptorchidism, Hypospadias, and Phthalate Load: A Large Case–Control Study

**DOI:** 10.3389/fphys.2018.00406

**Published:** 2018-04-24

**Authors:** Ravinder Anand-Ivell, Arieh Cohen, Bent Nørgaard-Pedersen, Bo A. G. Jönsson, Jens-Peter Bonde, David M. Hougaard, Christian H. Lindh, Gunnar Toft, Morten S. Lindhard, Richard Ivell

**Affiliations:** ^1^School of Biosciences, University of Nottingham, Nottingham, United Kingdom; ^2^Section of Neonatal Screening and Hormones, Department of Clinical Biochemistry and Immunology, Statens Serum Institut, Copenhagen, Denmark; ^3^Division of Occupational and Environmental Medicine, Department of Laboratory Medicine, Lund University, Lund, Sweden; ^4^Department of Occupational and Environmental Medicine, Bispebjerg Hospital, University of Copenhagen, Copenhagen, Denmark; ^5^Department of Clinical Epidemiology, Aarhus University Hospital, Aarhus, Denmark; ^6^Perinatal Epidemiology Research Unit, Department of Pediatrics, Aarhus University Hospital, Aarhus, Denmark; ^7^School of Veterinary Medicine and Science, University of Nottingham, Nottingham, United Kingdom

**Keywords:** INSL3, amniotic fluid, fetal steroids, cryptorchidism, hypospadias, phthalate, PFOS, testicular dysgenesis syndrome

## Abstract

The period of the first to second trimester transition in human pregnancy represents a sensitive window for fetal organogenesis, particularly in regard to the development of the male reproductive system. This is a time of relative analytical inaccessibility. We have used a large national biobank of amniotic fluid samples collected at routine amniocentesis to determine the impacts of exogenous endocrine disruptor load on specific fetal biomarkers at this critical time. While adrenal and testicular steroids are highly correlated, they are also mostly positively influenced by increasing phthalate load, represented by the metabolites 7cx-MMeHP and 5cx-MEPP, by perfluorooctane sulfonate (PFOS) exposure, and by smoking, suggesting an adrenal stress response. In contrast, the testis specific biomarkers insulin-like peptide 3 (INSL3) and androstenedione are negatively impacted by the phthalate endocrine disruptors. Using a case–control design, we show that cryptorchidism and hypospadias are both significantly associated with increased amniotic concentration of INSL3 during gestational weeks 13–16, and some, though not all steroid biomarkers. Cases are also linked to a specifically increased variance in the Leydig cell biomarker INSL3 compared to controls, an effect exacerbated by maternal smoking. No influence of phthalate metabolites or PFOS was evident on the distribution of cases and controls. Considering that several animal and human studies have shown a negative impact of phthalate load on fetal and cord blood INSL3, respectively, the present results suggest that such endocrine disruptors may rather be altering the relative dynamics of testicular development and consequent hormone production, leading to a desynchronization of tissue organization during fetal development. Being born small for gestational age appears not to impact on the testicular biomarker INSL3 in second trimester amniotic fluid.

## Introduction

Cryptorchidism and hypospadias represent two of the commonest congenital malformations in newborn boys, and both conditions have been linked to a hypothetical TDS (Skakkebaek et al., 2001; Wohlfahrt-Veje et al., 2009) implying a common etiology. This hypothesizes an insult of possible environmental, lifestyle, or pharmacological nature during a critical window of sensitivity in the transition from the first to the second trimester, a time soon after sex determination has occurred and the principal cellular components of the fetal testes are being established. Research in rodents has highlighted a role for Leydig cells and their major androgen products in both conditions, an opinion reinforced by the symptoms induced by some phthalate compounds administered at a comparable gestational time (Foster, 2006; Howdeshell et al., 2008; Wilson et al., 2008; Hu et al., 2009; Veeramachaneni and Klinefelter, 2014; van den Driesche et al., 2017). Recent work using human fetal testis explants and/or xenografts, however, has suggested that the human fetus may be less susceptible to phthalates than rodents (Albert and Jegou, 2014; Habert et al., 2014), and a recent systematic review and meta-analysis could show no epidemiological relationship between endocrine disrupting chemicals and male reproductive disorders (Bonde et al., 2017). Other studies point to a significant role of analgesic compounds, such as paracetamol, taken during pregnancy, in disrupting human fetal Leydig cell function (Kristensen et al., 2012; Mazaud-Guittot et al., 2013; Ben Maamar et al., 2017). In support of an effect of phthalates on the human male phenotype, epidemiological studies have indicated a significant association between maternal phthalate load and the androgen-dependent anogenital distance (Swan et al., 2005; Bornehag et al., 2015).

Besides being major producers of the androgens androstenedione and testosterone during this critical window in human pregnancy (Ivell et al., 2017), fetal Leydig cells are now known to make and secrete another major hormone, INSL3 (Ivell and Anand-Ivell, 2009). Genetic experiments in mice show that ablation of the gene encoding either INSL3 or its unique cognate receptor RXFP2 leads to bilateral cryptorchidism, due to a failure of the gubernacular ligament to expand, and thereby promote the first transabdominal phase of testicular descent (Nef and Parada, 1999; Zimmermann et al., 1999; Kamat et al., 2004). This role is supported by the treatment of mice during mid-gestation with an INSL3 antagonist (Yuan et al., 2010) leading to cryptorchidism. Furthermore, in mice treated gestationally with the estrogen diethylstilbestrol, there is both bilateral cryptorchidism and concomitant down-regulation of *Insl3* gene expression in the fetal testes (Emmen et al., 2000), a finding paralleled by the increased incidence of cryptorchidism amongst boys born of mothers similarly treated with diethylstilbestrol during pregnancy (Palmer et al., 2009). The common consensus is that whereas INSL3 is important for the first phase of testicular descent, fetal androgens are more important for the later inguino-scrotal phase of descent, as well as for the proper formation of the external genitalia (Hutson et al., 2013). However, it should be noted that androgens may be required for the induction of the INSL3 receptor, RXFP2 (Yuan et al., 2010), as well as for the relaxation and involution of the cranial suspensory ligament, which retains the indeterminate gonad in a peri-renal position (Lee and Hutson, 1999), implying that androgens are also required during the first transabdominal phase. Thus it appears that cryptorchidism and hypospadias might have common roots in a disruption of Leydig cell function and the production of INSL3 and androgens during early-mid gestation.

Part of the difficulty in unraveling the etiology of cryptorchidism is its highly heterogeneous manifestation (Hutson et al., 2013). Testes may be arrested in a high abdominal position or at any point before or within the inguinal canal. Cryptorchidism may be bilateral or more commonly unilateral. Moreover, it may also be transient, with a higher incidence of cryptorchidism occurring in newborns compared with 3 month-old infants; there is also a possibility for testes to re-ascend from the scrotum during childhood. Anatomically, particularly the first phase of testicular descent from a peri-renal position to the inguinal ring is complicated by the fact that this occurs with an opposing vector to other abdominal organs. This phase of descent occurs because the thickening of the gubernaculum under INSL3 influence effectively retains the fetal testes in the inguinal region while the remaining organs and tissues, including the kidneys, grow away in an antero-dorsal direction. Thus any factor which would cause a temporal discordance of this fetal growth trajectory could result in a ligament or a tissue being interposed in the normal path of movement of one or other testes, leading also to cryptorchidism (mostly unilateral; Hutson et al., 2013).

Regarding hypospadias, evidence points to this being largely a complication involving fetal androgen-deficiency (Kalfa et al., 2009), though it should be noted that androgens are also made in substantial amounts by the fetal adrenals of both sexes as well as by the testes (Wudy et al., 1999; Anand-Ivell et al., 2008; Scott et al., 2009; Ivell et al., 2017). Moreover, it is still not clear whether the human fetal testis is dependent upon an intact and functional HPG axis (and/or fetal hCG), or whether other factors may be involved in determining androgen production (Scott et al., 2009). For example, we know that the mouse fetal testes are largely independent of LH stimulation, rather being driven by ACTH as for the fetal adrenal (O’Shaugnessy et al., 2003). Moreover, one study suggests that human fetal Leydig cells at least up to 18 weeks are also independent of hCG (Word et al., 1989), though a more recent study indicates a clear stimulation of testosterone production by hCG (Hallmark et al., 2007).

One of the major difficulties in understanding the etiology of these two common congenital ailments is the fact that the critical period during human pregnancy which is implicated is also one of the experimentally least accessible. We and others have shown that amniotic fluid collected at routine genetic amniocentesis serves as a useful matrix by which to explore the endocrinology of the growing second trimester fetus (Wudy et al., 1999; Anand-Ivell et al., 2008). Most such samples are collected between gestational weeks 12 and 16, a time at which amniotic fluid still represents very much an exudate of fetal serum combined with other fluid components derived from the amniotic membranes (Underwood et al., 2005; Beall et al., 2007). Later in pregnancy, when the fetal skin has become keratinized, this simple relationship to fetal serum is probably no longer valid (Bacchi-Modena and Fieni, 2004). The present study represents the largest ever (425 controls; 421 cryptorchid; 109 hypospadias) case–control study to assess the role of fetal androgens and INSL3 in subsequent cryptorchidism and hypospadias, using second trimester amniotic fluid samples collected in Denmark as part of a national biobank (Jensen et al., 2012). Preliminary results from this study have already been published (Jensen et al., 2012, 2015; Toft et al., 2016); here we extend these analyses and specifically address those factors influencing Leydig cell function and which may be altered in subsequent cryptorchidism and hypospadias, and focusing on the critical window of sensitivity (weeks 13–16).

## Materials and Methods

### Study Population

Amniotic fluid samples were derived from a Danish biobank maintained at the State Serum Institute (SSI) in Copenhagen. Amniotic fluid samples, which had been collected at routine amniocentesis between 1980 and 1996, were selected from 25,105 samples from live-born singleton male offspring pregnancies which had complete obstetric data in the Danish Medical Birth Registry. Samples were centrifuged, and supernatants stored at -20°C until analysis. Main indications for amniocentesis were age (≥35 years) or increased risk of congenital malformation based on maternal serum analysis. The date of amniocentesis, date of birth, and estimated gestational age at birth were used to calculate the gestational week of amniocentesis (Jensen et al., 2012). Unique person identifiers were used to obtain offspring gender, gestational age at birth, parity, birth weight, and Apgar score (Knudsen and Olsen, 1998; Pedersen et al., 2006). All selected cryptorchid cases (*n* = 421; 1.64%) had a diagnosis of undescended testis according to the International Classification of Disease, as well as a corrective surgical procedure according to the Surgery and Treatment Classification of the Danish National Board of Health or the Nordic Classification of Surgical Procedures, as listed in the DNPR up to November 2008 (Lynge et al., 2011). To avoid possible inclusion of cases of iatrogenic cryptorchidism, boys with an inguinal hernia repair were excluded. The hypospadias group (*N* = 109; 0.43%) included all boys with that diagnosis in the DNPR. An equivalent number (*n* = 425) of controls were randomly selected from the 25,105 boys with complete medical entries in the DNPR. Full details of inclusion criteria are given in Jensen et al. (2015).

This study was approved by the Danish Regional Ethics Committee, the Danish National Board of Health, and the Danish Data Protection Agency.

### Analyses and Parameters

Insulin like peptide 3 was measured using a modified TRFIA as recently described (Anand-Ivell et al., 2013; Jensen et al., 2015). This assay used a sample volume of 0.1 ml and had a limit of detection of 10 pg/ml and inter- and intra-plate coefficients of variation of ≤8 and <1%, respectively. Because, it had previously been shown that INSL3 in amniotic fluid indicated a marked dependence on gestational age (Anand-Ivell et al., 2008), values were also converted to MoM to account for this additional variable factor (Lenke et al., 1989). Because of volume restrictions INSL3 could only be measured in 243 controls, 227 cryptorchids (bilateral and unilateral combined), and 73 hypospadias cases. The steroids androstenedione, testosterone, 17OH-progesterone, progesterone, DHEAS, and cortisol were all assayed using LC/MS–MS as previously described (Jensen et al., 2015). Cotinine concentration, as an index for acute maternal exposure to tobacco smoke, was measured as previously (Jensen et al., 2015), with smokers defined as those women with amniotic fluid levels >85 ng/ml, and passive smokers as those with levels 85 > 25 ng/ml (Jauniaux et al., 1999). Non-smokers had <25 ng/ml cotinine. Fetuses were defined as SGA or not using the formula of Marsal et al. (1996). This calculation is based on ultrasound observations of uncomplicated pregnancies from four Scandinavian centers and calculates SGA as >2 standard deviations below the mean corrected birth weight. The two major phthalate metabolites 5cx-MEPP deriving from exposure to DEHP and 7cx-MMeHP, deriving from DiNP, as also PFOS were measured by LC/MS–MS as previously described in detail (Jensen et al., 2012; Toft et al., 2016) with limits of detection at 0.05, 0.02, and 0.02 ng/ml, respectively. Sample numbers available for measuring 5cx-MEPP, 7cx-MMEHP, and PFOS across all gestational ages were 300, 270, and 75 for controls, cryptorchids, and hypospadias cases, respectively, and for weeks 13–16, 190, 146, and 48; for measuring steroids across all gestational ages, N was 258, 237, and 79 for controls, cryptorchids, and hypospadias cases, respectively, and for weeks 13–16, the corresponding figures were 164, 123, and 51.

Many of the samples measured had been stored for up to 30 years at -20°C prior to analysis. They might thus have suffered evaporation/sublimation and/or analyte degradation in that time. To check this, measured parameters were analyzed against both date of sample collection and total volume of stored sample. No significant trends for any analyte were identified (Jensen et al., 2012). For INSL3 we have shown that serum samples stored at -20°C for at least 5 years remained stable with no significant difference in INSL3 content (data not shown), and previously we have shown that human amniotic fluid samples maintained INSL3 content over several freeze-thaw cycles and when left at room temperature for 24 h without significant change (Anand-Ivell et al., 2008).

### Statistical Analysis

Data were analyzed using GraphPad Prism version 7 or the SPSS package (for multiple correlation analysis). For some analyses, data were grouped according to week of amniocentesis, where for example week 16 represented all samples from 16+0 to 16+6. Column statistics were assessed by Levene’s test (Levene, 1960), as described in SPSS, to check for equality of variance. Where variance was shown to be unequal, comparisons used the Mann–Whitney non-parametric test; otherwise significance was estimated by ANOVA followed by Newman–Keuls or Student’s *t post hoc* tests. Continuous variables were log-transformed prior to statistical analysis. Multiple correlation analysis used untransformed data and was corrected against gestational week of amniotic fluid sample as confounder, since several parameters showed significant trends across gestation (**Supplementary Figure S1**).

## Results

### Amniotic Fluid INSL3 and Gestational Age at Amniocentesis

**FIGURE 1:**
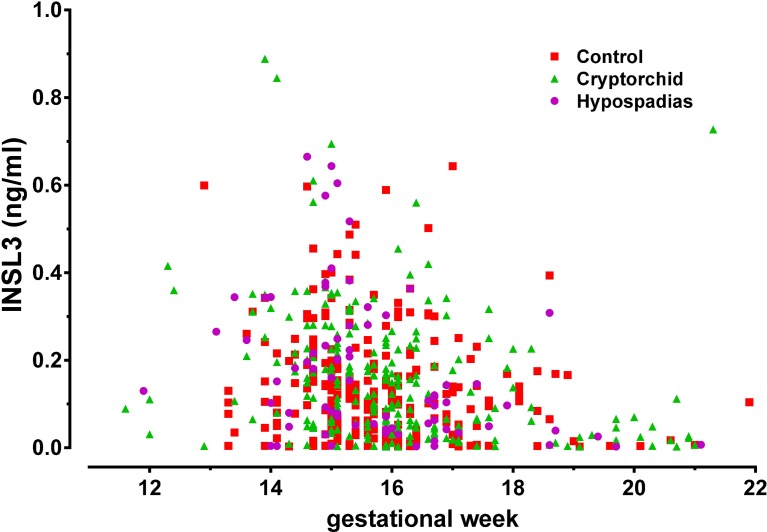
Scatter-plot of human amniotic fluid INSL3 concentration for all available samples from normal controls (red squares), combined cryptorchid cases (green triangles), and hypospadias cases (purple circles), plotted against gestational week of amniocentesis.

**FIGURE 2:**
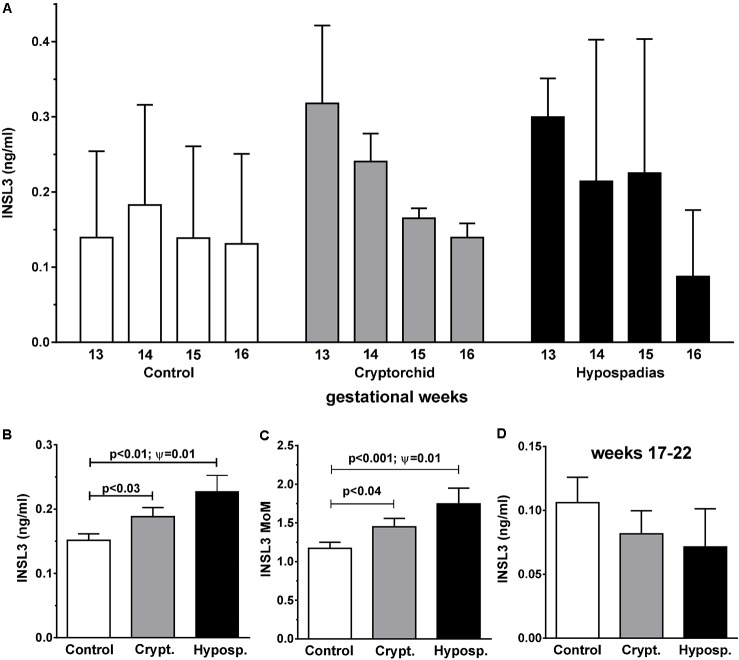
Frequency distributions of amniotic fluid INSL3 concentration (means + SEM) collected at gestational weeks 13–16 for controls, combined cryptorchids and hypospadias cases **(A)**. Since individual weeks between 13 and 16 were not significantly different from one another for cases and controls, these were combined in **(B)** (expressed as absolute concentration) and **(C)** (expressed age-corrected as MoM). **(D)** As in **(B)** but for gestational weeks 17–22. Differences between means were significant only where indicated. Ψ Represents significance for Levene’s test for differences in variance.

**Figure 1** shows the distribution of INSL3 concentration in amniotic fluid plotted against gestational age at amniocentesis for controls (red squares), cryptorchid (green triangles), and hypospadias (purple circles) cases. For cryptorchids, unilateral, bilateral and undefined cases were combined, since these showed no significant differences for any parameter measured. All data appear to fit a common distribution with neither cases nor controls appearing to depart from this pattern, which closely follows what we had shown previously for a smaller collective of United States subjects (Anand-Ivell et al., 2008). There is a clear maximum between gestational weeks 13 and 16, with most values declining rapidly after that time. Closer inspection, however, reveals that for all cases compared to controls there appears to be skewness in the data suggesting higher INSL3 values for cases in the earlier weeks of gestation (**Figure 2A**). Since there are no significant differences in INSL3 values within groups between weeks 13 and 16, for which these concentrations represent maximum values, these results can be pooled to provide the summary **Figure 2B**. This panel shows that in the early second trimester, when amniotic fluid INSL3 is maximal, both cryptorchid and hypospadias cases have significantly elevated INSL3 concentration compared to controls. The mean INSL3 concentration for the control subjects is similar to what had been shown previously for United States male pregnancies at this gestational time (ca. 0.10 ng/ml; Anand-Ivell et al., 2008), as well as for a smaller Danish set collected with a much shorter storage time (ca. 0.12 ng/ml; Bay et al., 2008).

When INSL3 values for weeks 13–16 are corrected for gestational age by converting to MoM, we still observe a significant difference in the mean values for controls versus both cryptorchids and hypospadias cases (**Figure 2C**). There is also a significant difference in the variance between the cryptorchid (unilateral + bilateral) and the control groups for this data-set, as well as between the hypospadias and control groups, assessed using Levene’s test.

We also compared the residual INSL3 concentrations in weeks 17–21 for cases and controls (**Figure 2D**). Although the uncorrected mean values are reduced in both cryptorchid and hypospadias cases compared to controls, this does not reach statistical significance, probably due to the large proportion of samples at or close to the assay limit of detection. Converting to MoM does not alter this (not shown).

### Amniotic Fluid INSL3 and Maternal Smoking

**FIGURE 3:**
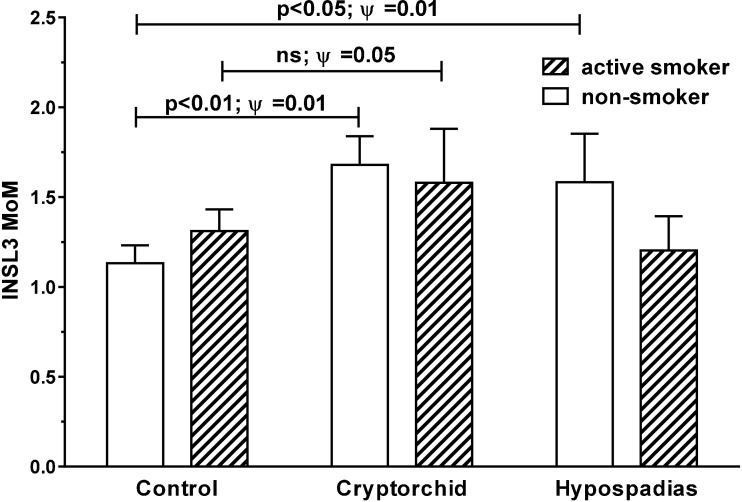
Age-corrected INSL3 MoM values for all gestational weeks combined for cases and controls, separated into active smokers (cotinine > 85 ng/ml) and non-smokers (cotinine < 25 ng/ml). Passive smokers (cotinine 25 < 85 ng/ml) were intermediate and omitted for clarity. Significant differences between means were only evident amongst non-smokers. Ψ Represents significance for Levene’s test for differences in variance. ns, not significant.

Comparing amniotic fluid samples for smokers, passive smokers, and non-smokers (cotinine >85, 25–85, and <25 ng/ml, respectively) for INSL3 MoM values for all gestational ages, shows no differences, when all cases and controls are pooled (data not shown). However, when separated out into cases versus controls (**Figure 3**), INSL3 MoMs are significantly greater (*p* < 0.003) in the cryptorchid group for non-smokers versus controls, though just not significant for smokers (passive smokers are intermediate between smokers and non-smokers and are omitted here for clarity). Both smokers and cases (cryptorchid/hypospadias) indicate a higher overall variance in INSL3 MoM, thus accounting for the failure to achieve significance in the absolute differences. Correlation analysis shows no relationship between individual amniotic cotinine concentration and either INSL3 concentration or INSL3 MoM values in cases or controls or in both combined, nor when data are restricted to weeks 13–16 (**Supplementary Tables S1**–**S5**).

### Amniotic Fluid INSL3 and Fetal Size for Gestational Age

**FIGURE 4:**
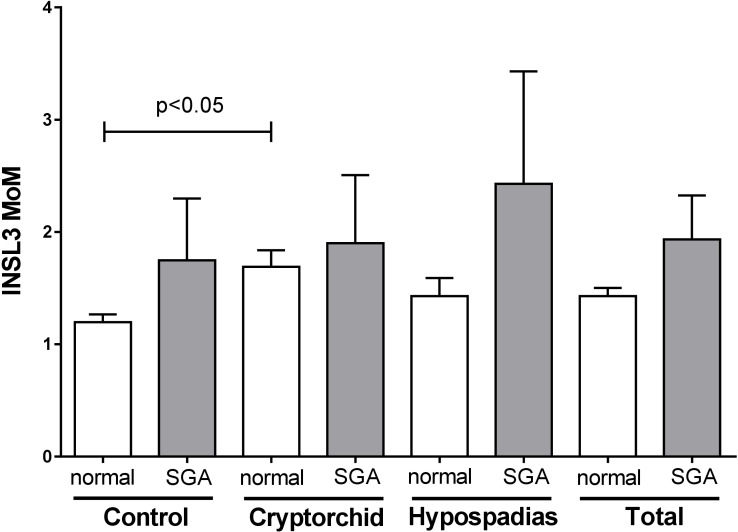
Age-corrected INSL3 MoM values for all gestational weeks combined for cases and controls, separately and together (total), separated into pregnancies calculated to be SGA and normal pregnancies (not SGA). Only the difference between non-SGA controls and cryptorchids was considered significantly different.

In order to determine whether amniotic fluid INSL3 concentration might be related to intrauterine growth, we applied the formula of Marsal et al. (1996) in order to identify those fetuses born SGA. There was a higher proportion of such SGA pregnancies in both cryptorchid (9.3%) and hypospadias (8.2%) groups, compared to the controls (4.9%). Pregnancies identified as SGA were then compared with non-SGA normal pregnancies in regard to amniotic INSL3 concentration, comparing cases and controls pooled, as well as individually (**Figure 4**). Other than the reported increased variance for cryptorchids, there appeared to be no significant impact on the INSL3 MoM of being born SGA. The same was also true for absolute INSL3 concentrations restricted to weeks 13–16. We also see no relationship between INSL3 expression and gestational age at birth (data not shown).

### Amniotic Fluid INSL3 and Steroid Concentrations

**FIGURE 5:**
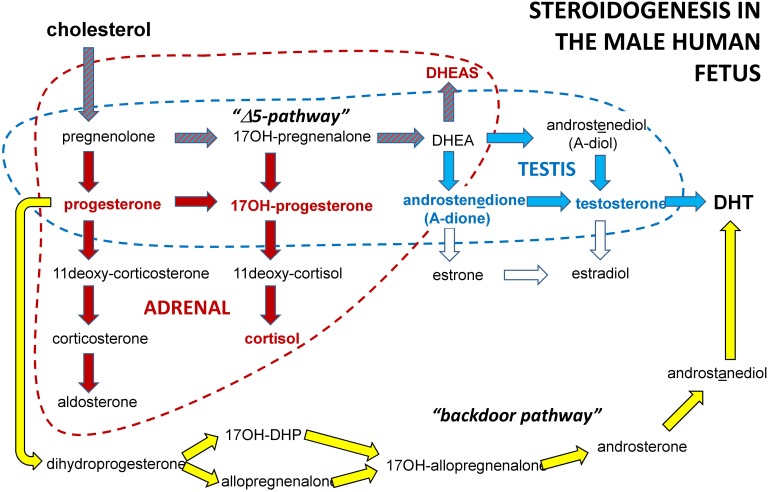
Schematic to illustrate the principal steroidogenic pathways in the male human fetus for both testes and adrenals. In bold type (red or blue) are indicated those steroids used here as biomarkers of fetal steroidogenesis.

For male pregnancies, steroids measured in amniotic fluid derive from the fetal testis, the fetal adrenal gland, and in small part from the placenta (**Figure 5**; Ivell et al., 2017). Comparisons between amniotic fluid steroids from male and female fetuses (Wudy et al., 1999; Anand-Ivell et al., 2008) indicate that testosterone and androstenedione are predominantly of testicular origin, being enriched for male fetuses. In contrast, progesterone, 17OH-progesterone, DHEAS and cortisol do not differ in relation to fetal gender (Wudy et al., 1999), and are thus predominantly of adrenal and/or placental origin. Not surprisingly, because of their common metabolic pathways, all measured steroids are highly correlated to one another (*p* < 0.001), when measured across gestation for cases and controls combined (**Supplementary Table S1**). Absolute concentrations of the various steroids measured in amniotic fluid show that androstenedione (2–10 ng/ml; 5–95% confidence limits) is 3–4 fold higher than testosterone (0.5–3.0 ng/ml), and comparable in amount to 17OH-progesterone (4–10 ng/ml). Cortisol and DHEAS are greater again (5–100 ng/ml) and illustrate the larger steroidogenic capacity of the adrenal gland at this time of gestation. Progesterone (also of placental/luteal origin and a major steroid precursor in all pathways) is highest at 100–300 ng/ml concentration. Comparing steroid concentrations across gestational weeks, there is no significant effect of gestational age (weeks 11–21) on androstenedione, testosterone, or progesterone. For cortisol, 17OH-progesterone and DHEAS there is a small but significant trend for increasing concentration in amniotic fluid between weeks 11 and 21 (**Supplementary Figure S1**), but only for cortisol is this trend evident between the critical weeks 13–16.

Androstenedione, 17OH-progesterone, DHEAS and cortisol, though not testosterone or progesterone, are positively correlated (*p* < 0.02) with cotinine levels (**Supplementary Table S1**; all cases and controls combined), implying that maternal smoking is promoting increased steroidogenesis.

**FIGURE 6:**
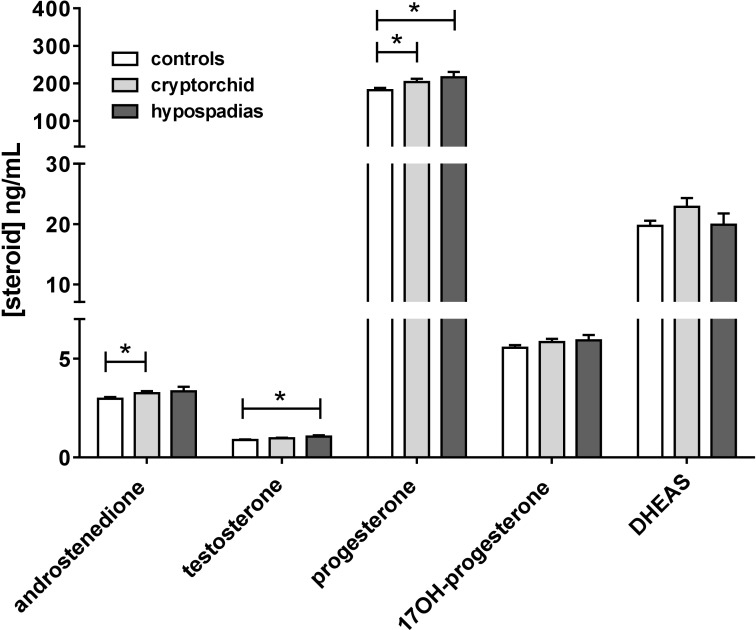
Mean steroid concentrations in amniotic fluid combined across all gestational ages, separated into normal controls, combined cryptorchid and hypospadias cases. ^∗^*p* < 0.05.

Comparing all cases and controls for all steroids measured and over all gestational ages, showed small but significant differences for testosterone (*p* < 0.05) and for progesterone (*p* < 0.05) for hypospadias cases versus controls, where for both steroids these were mildly elevated (**Figure 6**). Cryptorchid cases were significantly elevated compared to controls for progesterone and also for androstenedione (**Figure 6**). However, when only weeks 13–16 are considered, then probably because of the reduced sample size only testosterone is significantly different (18% increased; *p* < 0.05) between cases and controls, and then only for hypospadias cases (**Supplementary Figure S2**).

It should be noted that because of lack of sufficient sample availability, it was not possible to measure all parameters in all samples. Thus only where both INSL3 and all measured steroids were available for the same amniotic fluid samples could multiple correlation analysis be carried out. As might be anticipated for major Leydig cell products and comparing samples from all gestational weeks, there is a statistically positive correlation between INSL3 MoM and in order of significance, testosterone (*p* < 0.001), androstenedione (*p* = 0.005), and DHEAS (*p* = 0.043), for cases and controls combined, and corrected for gestational week (**Supplementary Table S1**). For controls only (**Supplementary Table S2**), there is a positive correlation for androstenedione (*p* = 0.007) and testosterone (*p* = 0.04). Amongst cryptorchids (**Supplementary Table S3**), a significant positive correlation with INSL3 MoMs is observed only for testosterone (*p* < 0.001), although INSL3 concentration itself also correlates strongly with androstendione (*p* = 0.005). No correlations were observed for INSL3 amongst the hypospadias cases (**Supplementary Table S4**).

As explained earlier, INSL3 concentrations limited to weeks 13–16 are more informative, representing maximal and physiologically relevant INSL3 expression. When steroids are now compared, we see a significant positive correlation between INSL3 MoMs (as also INSL3 concentration) and both androstenedione (*p* = 0.01) and testosterone (*p* < 0.01; **Supplementary Table S5**).

### Influence of Xenobiotic Compounds

The present study has assessed the amniotic fluid concentration of 5cx-MEPP as the chief metabolite of DEHP, and 7cx-MMeHP as the main metabolite of DiNP, as well as the fluorinated compound PFOS. Firstly, both 5cx-MEPP, and 7cx-MMeHP, showed small but significant trends to increase across gestation (weeks 11–22; cases and controls combined; **Supplementary Figure S1**). Only 7cx-MMeHP showed a small but significant (*p* < 0.05) correlation (negative) with cotinine levels (**Supplementary Table S1**; cases and controls combined; **Supplementary Table S2**; controls only). Importantly, both phthalate metabolites were positively correlated amongst each other (*p* < 0.001; cases and controls combined; **Supplementary Table S1**). 5cx-MEPP was also highly positively correlated (*p* < 0.5 to *p* < 0.001; **Supplementary Table S1**) with all steroids measured, except for progesterone (not significant). In contrast, cx7-MMeHP was correlated only with androstenedione (*p* < 0.02; negatively) and DHEAS (*p* = 0.002; positively).

Comparing cases with controls across the gestational range, there was no significant difference in mean or variance for either of the measured phthalate metabolites (not shown). When samples are restricted to the window of sensitivity for testis development in weeks 13–16, there is also no significant difference between cases and controls (**Supplementary Figure S2**).

When we compare INSL3 values for weeks 13–16 as representative of Leydig cell functionality at this critical time, then there is a negative correlation (*p* = 0.04) with 7cx-MMeHP for controls only (not shown), as well as cases and controls combined (**Supplementary Table S5**), and a similar negative correlation (*p* < 0.04) with 5cx-MEPP for cases and controls combined (**Supplementary Table S5**). There is no correlation between INSL3 and any phthalate metabolite within the cryptorchid and hypospadias cases alone at this restricted time window in gestation (not shown).

The environmental pollutant PFOS was significantly positively correlated with all steroids measured (cases and controls combined; all gestational weeks) as well as with the phthalate metabolites 7cxMMEHP (**Supplementary Table S1**). PFOS was not correlated with INSL3 MoM values across gestation (weeks 11–21; **Supplementary Table S1**), nor when data were restricted to gestational weeks 13–16 (**Supplementary Table S5**). As for the phthalate metabolites, also PFOS exposure showed no statistically significant differences between cases and controls within the critical window of weeks 13–16 (**Supplementary Figure S2**).

None of the above significant relationships was altered when multiple correlation analysis was additionally corrected for either cotinine and/or PFOS concentration as possible confounders (not shown).

## Discussion

Cryptorchidism and hypospadias are amongst the commonest malformations in newborn infants representing, respectively, 2–9 and 0.2–0.7% (Main et al., 2010) of all male births, and are considered by some to be symptoms of a common TDS (Wohlfahrt-Veje et al., 2009). Such disturbances of sexual development in male human offspring are thought to have their origins relatively early during pregnancy, soon after SRY-driven sex determination and during early testicular organogenesis. This corresponds approximately to the end of the first trimester and the early second trimester of pregnancy, though recent studies using human fetal testis explants suggest weeks 8–10 as those most sensitive in this regard to the analgesic ibuprofen (Ben Maamar et al., 2017). Our understanding of the etiology of these conditions derives largely from animal experimentation as well as from sporadic genetic mutations in the human population. Whilst there is an obvious role here for fetal testicular androgens, we still know relatively little about how these and other factors influence early stages of the relevant organogenesis. Recently and largely based on animal experimentation, there is concern that maternal exposure to certain endocrine disrupting chemicals in the environment may be exacerbating the incidence of such disorders (Diamanti-Kandarakis et al., 2010; Juul et al., 2014), though a recent epidemiological meta-analysis failed to identify such a relationship (Bonde et al., 2017).

Unfortunately, the highlighted critical period of early pregnancy is also a time in the human which is analytically relatively inaccessible, with assessment of pollutants and/or end-point physiology relying heavily on indirect measurement, e.g., maternal urine, breast milk, cord blood hormones, perinatal, juvenile, or even adult phenotype. Amniocentesis is a procedure whereby a sample of amniotic fluid and contained fetal cells is removed by abdominal needle puncture. It is routinely offered to older women, or where a genetic risk is suspected, during weeks 12–20 of pregnancy for fetal genetic analysis. The resulting amniotic fluid supernatants thus represent a unique opportunity to explore human fetal metabolism at an otherwise relatively inaccessible period of pregnancy. In the present study we have taken advantage of the largest known biobank of preserved amniotic fluid samples to assess known markers of fetal testis physiology within the sensitive window of development and compare these in a case–control format, together with known sentinel ED substances, to determine how these might be related at this critical period during human gestation.

Of the biomarkers assessed, only INSL3 is absolutely specific for the steroid-producing Leydig cells of the fetal testis (Ivell and Anand-Ivell, 2009). Because of this specificity of expression at a time when many organ systems are actively developing, INSL3 has been considered an important biomarker for more general fetal organogenesis at this time (Anand-Ivell and Ivell, 2014). Amongst the steroids, both androstenedione and testosterone are relatively specific for the fetal testis, though are also produced by the fetal adrenal gland which is very active at this time in pregnancy. Of the other steroids measured, these are largely products of the fetal adrenal gland (Ivell et al., 2017). INSL3 is responsible for the first transabdominal phase of testicular descent and is thus directly implicated in cryptorchidism. Activated androgen receptors are involved both in cryptorchidism (early and late stages) as well as in hypospadias and associated malformations. In this context it is important to note that whilst fetal testosterone is a very important androgen, androstenedione also acts as a weak androgen (Chen et al., 2004), and can additionally activate estrogen receptors alpha and beta (Miller et al., 2013), which may also be involved in gonadogenesis (Cederroth et al., 2007). However, testosterone itself is less important than its metabolite DHT, which is produced locally in target tissues. Recently, it has been shown also for the human fetus, that significant amounts of DHT can be generated in the fetal testis by the so-called ‘back-door’ pathway, which avoids testosterone (Fukami et al., 2013; **Figure 5**). Thus whilst the biomarkers selected in this study are likely to reflect well aspects of testicular (in particular Leydig cell) physiology, they may not be fully informative about factors responsible for the etiology of cryptorchidism and hypospadias.

Reflecting its high cell-type and fetal specificity, amniotic INSL3 concentration is most informative as a biomarker of Leydig cell function. However, a factor of concern is the high number of samples with low or undetectable INSL3 concentration. Similar observations were made previously (Anand-Ivell et al., 2008). There is no evidence for any loss of INSL3 during storage, or repeated freeze-thaw cycles, and the TRFIA assay itself is very robust and reliable. The INSL3 concentration may be reflecting the dynamic nature of amniotic fluid at this time in early pregnancy when its volume relationship to fetal serum is changing dramatically, and its source is probably also changing (Anand-Ivell and Ivell, 2014). Studies in the cow, pig, and rat suggest that amniotic fluid samples in mid-late gestation represent between 1-3rd and 1-10th of the INSL3 concentration in fetal serum at that time (Anand-Ivell et al., 2011; Anand-Ivell and Ivell, 2014; Vernunft et al., 2016). Thus while maximal INSL3 levels, as are evident between weeks 13 and 16, will depend on Leydig cell numbers and differentiation status, the subsequent decline in INSL3 concentration may be less due to a fall in production as to the changing fluid dynamics of the amniotic compartment. It should also be noted that amniotic fluid volume itself changes markedly across gestation from approximately 10 ml at gestational weeks 8–9, to about 100 ml at week 12, increasing to nearly 400 ml by week 18 (Brace and Wolf, 1989; Falcon et al., 2005; Rolo et al., 2010; Sandlin et al., 2014). More importantly, the individual variation in amniotic fluid volume at any one time point is also large (up to 10-fold between the 25^th^ and 75^th^ percentile; Sandlin et al., 2014). Thus a change in amniotic hormone concentration can be as much due to a specific change in hormone production as to a change in amniotic fluid volume. It should be noted that significantly reduced amniotic fluid volume (oligohydramnios) is associated with utero-placental insufficiency and fetuses born SGA (Morris et al., 2014). Unfortunately, no information regarding amniotic fluid volume was available in the present retrospective study. In a previous study (Anand-Ivell et al., 2008) ultrasound estimates of amniotic fluid volume did not appear to vary in normal pregnancies.

Comparing the cryptorchid and hypospadias cases with controls, particularly within the physiologically informative weeks 13–16, indicates firstly, a small mean increase of INSL3 concentration in both cryptorchid and hypospadias cases. This had not been noted in the earlier analysis which made use of a logistic regression analysis using tertiles from all gestational ages (Jensen et al., 2015). Secondly, there appears to be a significantly increased variance within these populations in cases compared to controls, particularly noticeable in non-smokers. Thirdly, there is an altered distribution, with the higher INSL3 values amongst the cases occurring at an earlier gestational time-point. Interestingly, looking at the later time-points in gestation (weeks 17–21) suggests (though not significantly) that later in gestation INSL3 concentration becomes reduced in cases versus controls, thus lending support to the recent studies indicating a reduced INSL3 concentration in the cord blood of infants with cryptorchidism (Bay et al., 2007; Fenichel et al., 2015). Taken together, these data imply an altered Leydig cell functionality in the cases compared to controls, these cells possibly responding earlier and more vigorously to the conditions which eventually lead to the pathologies. There is no evidence for a reduced Leydig cell function in these cases compared to controls. All subjects can be considered as representative of the general population; this differs therefore from the more extreme pharmacological situations in humans or rodents, for example, where high diethylstilbestrol or phthalate concentrations can lead to a high incidence of cryptorchidism with accompanying INSL3 depletion (Emmen et al., 2000; McKinnell et al., 2005; Palmer et al., 2009). A similar significant increase in INSL3 expression was also noted in the previous, smaller study of United States amniotic fluid samples (Anand-Ivell et al., 2008) in relation to preeclampsia. Moreover, a study of INSL3 in rats during puberty suggested that one of the effects of maternal treatment with dibutyl phthalate or diethylstilbestrol was to cause a precocious shift in the dynamics of INSL3 expression, with the pubertal increase in hormone expression being advanced upon maternal ED treatment (Ivell et al., 2013). This appeared to be due to altered relative rates of Leydig cell proliferation and differentiation.

There is no evidence for an influence of phthalate metabolites or PFOS within the normal ranges of human exposure on the incidence of cryptorchidism or hypospadias, even when analysis is restricted to the sensitive window between weeks 13 and 16.

When looking at data from all available weeks of gestation, both testosterone and progesterone also appear to be elevated compared to controls in hypospadias samples both as mean, but also in their variance (Levene’s test). Similarly, androstenedione and progesterone are both slightly elevated in cryptorchid cases. This effect is not apparent when assessing steroids only within weeks 13–16 of pregnancy; here only testosterone is significantly elevated, and then only in the hypospadias cases compared to controls. This is an important finding, since it would tend to refute the alternative explanation that any increases in the concentration of amniotic hormones in cases compared to controls might be due to a significantly reduced amniotic fluid volume. These findings reinforce the notion that Leydig cell function may indeed be physiologically different in cases compared to controls, possibly over-responding to some external ‘stress’ factors. The increased variance is particularly interesting since it implies that in this large population there are an increased number of discrepant testes, some of which may be under-producing, many of which may be over-producing, compared to controls. Altogether the results indicate a larger ‘at risk’ population, more likely to be impacted by other confounding factors, such as maternal smoking. The fact that INSL3 and androstenedione (both Leydig cell biomarkers) are closely correlated in the weeks 13–16 samples (cases and controls combined) and that also androstenedione shows an increased variance (Levene’s test), if not mean, in the hypospadias cases reinforces this notion, that there is a larger ‘at risk’ population amongst the cases compared to controls. None of the adrenal steroids show similar patterns of significantly altered mean or variance between cases and controls. The notion of increased variance (as opposed to or in addition to increased mean) for a parameter linked to pathology is not new. It is similar in a way to the concept of “superfertility” that is reported to be associated with at-risk pregnancies (Teklenburg et al., 2010) or in the positive fertility effects of being a “quiet” embryo (Leese et al., 2007).

Of possible extrinsic factors that might be involved in altered Leydig cell function, maternal smoking and ED load are likely to be relevant. It should be noted that of the ED substances tested, both PFOS and the DEHP metabolite 5cx-MEPP indicate a positive correlation with almost all steroids, including the androgens, measuring all samples across gestation. Interestingly, only the DiNP metabolite cx7-MMeHP, as well as the DEHP metabolite 5cxMEPP showed significant negative correlations, but only for INSL3 and androstenedione in this sample population (**Supplementary Tables S1–S5**), whereas PFOS and cotinine showed no specific interactions with INSL3. In other words, only the specific Leydig cell biomarkers INSL3 and androstenedione were negatively impacted by putative phthalate metabolites. Otherwise, these, like PFOS and cotinine, had rather a positive impact on steroidogenesis, most of which occurs in the fetal adrenal gland, suggesting an increased fetal stress response due to the increased ED load. It is to be noted that the results presented here have mostly used partial correlation analysis corrected for gestational age, whereas an earlier analysis of the same data, which showed a significant negative effect of PFOS on INSL3 concentration, had corrected for additional confounders and transformed the PFOS and hormone data prior to logistic regression analysis using tertiles (Jensen et al., 2015; Toft et al., 2016). Here, our analysis also indicated a weak negative relationship between PFOS and INSL3 for weeks13–16 (**Supplementary Table S5**), though this failed to achieve significance.

Cotinine, as an indicator of maternal smoking was generally positively correlated with most steroids, when cases and controls were combined across gestation. Similar findings have been shown in the adult human, where smoking is linked to increased circulating testosterone, though to decreased INSL3 (Atlantis et al., 2009). In the present study there is no direct association of any kind between cotinine levels and INSL3. However, cotinine, being a relatively short-lived metabolite, is representative only of acute rather than chronic smoking and hence is less likely to correlate with differentiation-dependent parameters.

Finally, we assessed the possible impact of fetal growth on the various biomarkers, using a calculated estimate of SGA. Altogether, 7.2% of all pregnancies could be identified as SGA. Amongst these SGA pregnancies, and as suggested by others (Main et al., 2006), there appeared to be an increased risk of hypospadias and cryptorchidism (together 9% of all cases compared to 5% of control pregnancies), although none of the measured biomarkers, including INSL3, showed any association (negative or positive) with SGA. This thus appears to differ from an earlier, smaller study of INSL3 in amniotic fluid, where INSL3 values were shown to be significantly associated with age-corrected birth weight (Anand-Ivell et al., 2008).

Taken together, the results from this first large case–control study of amniotic fluid biomarkers, collected during the sensitive gestational window for testicular organogenesis, shows that particularly Leydig cell biomarkers appear to be increased, rather than decreased as might have been expected, in cryptorchidism and hypospadias. Significantly also, the variance about these biomarkers is also increased, strongly suggesting that at a population level Leydig cell functionality is more likely to be impaired or at risk to other confounding factors in the cases compared to controls. Moreover, when attempting to identify possible factors with a negative impact on Leydig cell functionality, the phthalate metabolites 5cx-MEPP or 7cx-MMeHP indicated the requisite negative correlation with INSL3 and/or androstenedione, without, however, implicating these compounds directly in the etiology of cryptorchidism or hypospadias. This agrees with a recent quantitative study on the impact of INSL3 and testosterone reduction on phenotype in rats, wherein it is suggested that >40% reduction in either hormone (or its mRNA) is required before there is evidence of any phenotypic impact (Gray et al., 2015). Recently, several studies have shown that INSL3 measured in cord blood, i.e., substantially later than its expression maximum in the fetus, is significantly reduced in cryptorchid cases compared to controls (Bay et al., 2007; Fenichel et al., 2015). Taken together, these data support the notion that cryptorchidism and/or hypospadias appear to be associated with a shift in the dynamics of INSL3 expression in the fetus, with precocious hormone expression and presumably Leydig cell differentiation in cases compared to controls. Thus INSL3, possibly as a result of exposure to EDs, such as phthalates, would increase earlier and decline sooner, and thus be out of synchrony with other ongoing morphological processes, thereby encouraging cryptorchidism and/or hypospadias.

## Author Contributions

RA-I, RI, BN-P, ML, and J-PB conceptualized and funded this study. BN-P, DH, and AC sorted and retrieved amniotic fluid samples from the biobank and performed the steroid assays. BJ and CL carried out the assays for environmental contaminants. RA-I and RI were responsible for the conception and writing of this article, for the measurement of INSL3, and for the statistical analysis presented here. CL, GT, and J-PB performed data management and reported to the major financial contributors. All authors critically revised previous versions of this manuscript, and approved the final version for submission.

## Conflict of Interest Statement

The authors declare that the research was conducted in the absence of any commercial or financial relationships that could be construed as a potential conflict of interest.

## Supplementary material

FIGURE S1 Regression analyses across gestational age for amniotic fluid levels of **(A)** DHEAS, **(B)** cortisol, and **(C)** 17OH-progesterone. All other steroids measured showed no significant age-related change. **(C–E)** Represent regression analyses for PFOS, and for the phthalate metabolites 5cx-MEPP and 7cx-MMeHP, respectively. The outlined boxes indicate those samples lying within the testicular window of sensitivity, weeks 13–16.

FIGURE S2 Mean steroid and environmental xenobiotic concentrations in amniotic fluid for weeks 13–16, separated into normal controls, combined cryptorchid and hypospadias cases. ^∗^*p* < 0.05.

TABLE S1 Cases and controls combined (weeks 11–22; corrected for gestational week).

TABLE S2 Controls only (weeks 11–22; corrected for gestational week).

TABLE S3 Cryptorchid only (weeks 11–22; corrected for gestational week).

TABLE S4 Hypospadias only (weeks 11–22; corrected for gestational week).

TABLE S5 Cases and controls combined (weeks 13–16; corrected for gestational week).

## Abbreviations

5cx-MEPP: mono(2-ethyl-5carboxypentyl) phthalate
7cx-MMeHP: mono(4-methyl-7-carboxyheptyl) phthalate
ACTH: adrenocorticotropic hormone
DEHP: di(2-ethylhexyl) phthalate
DHEAS: dehydroepiandrosterone sulfate
DHT: dihydrotestosterone
DiNP: diisononyl phthalate
DNPR: Danish National Patient Registry
ED: endocrine disruptor
hCG: human chorionic gonadotropin
HPG: hypothalamo-pituitary-gonadal
INSL3: insulin-like peptide 3
IUGR: intrauterine growth restriction
LC/MS–MS: liquid chromatography with tandem mass spectrometry
LH: luteinising hormone
MoM: multiple of the median
PFOS: perfluorooctane sulfonate
RXFP2: relaxin family peptide receptor 2
SGA: small for gestational age
TDS: testicular dysgenesis syndrome
TRFIA: time resolved fluorescent immunoassay
