# Supplementary material for: Amniotic Fluid INSL3 Measured During the Critical Time Window in Human Pregnancy Relates to Cryptorchidism, Hypospadias, and Phthalate Load: A Large Case–Control Study
Source: Front Physiol. 2018 Apr 24;9:406. doi: 10.3389/fphys.2018.00406 (PMC5928321; doi:10.3389/fphys.2018.00406)
Supplement: FIGURE S1 — Regression analyses across gestational age for amniotic fluid levels of (A) DHEAS, (B) cortisol, and (C) 17OH-progesterone. All other steroids measured showed no significant age-related change. (C–E) Represent regression analyses for PFOS, and for the phthalate metabolites 5cx-MEPP and 7cx-MMeHP, respectively. The outlined boxes indicate those samples lying within the testicular window of sensitivity, weeks 13–16. [file Presentation_1.PDF]

Suppl Fig 1

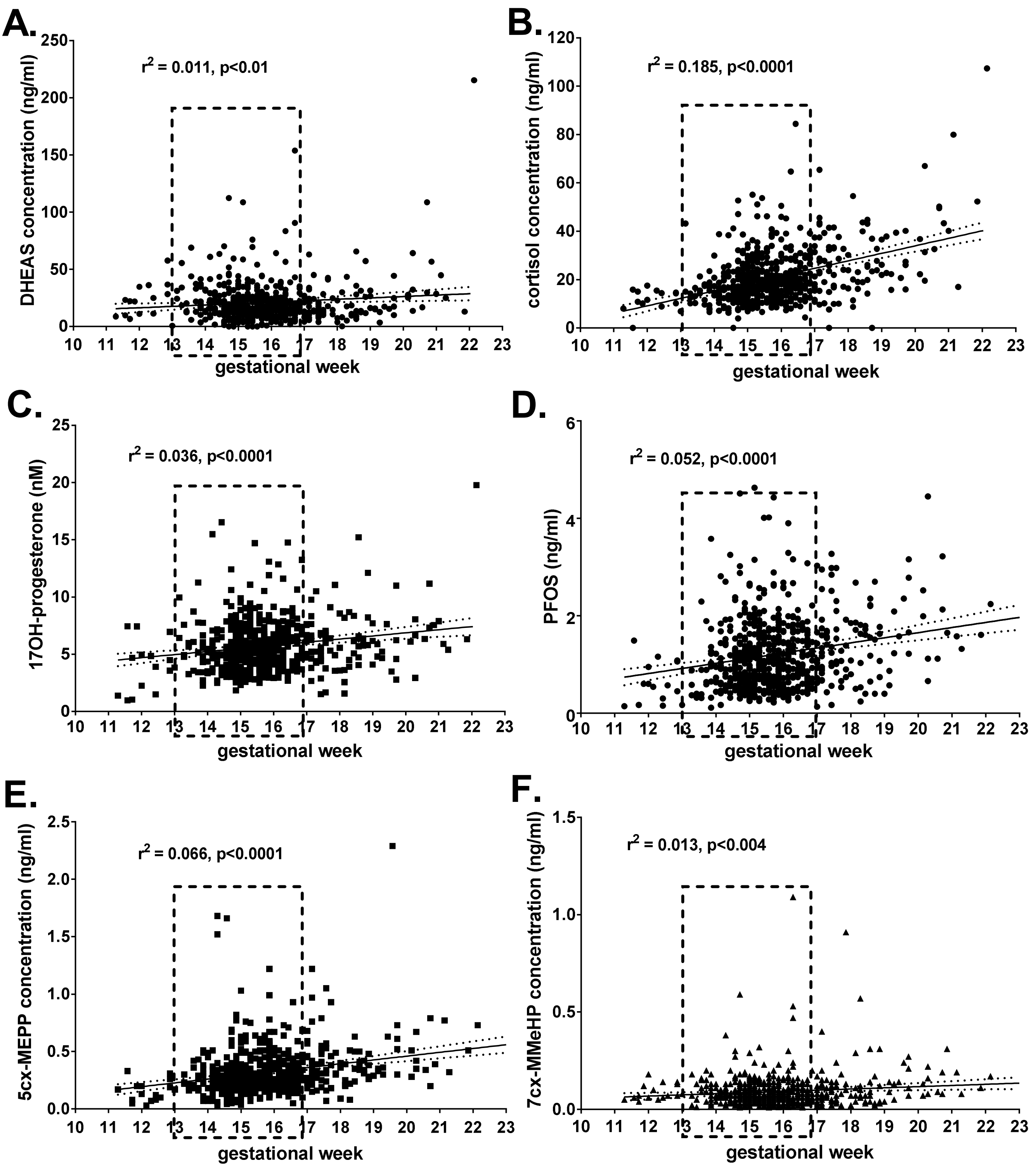
A.
DHEAS concentration (ng/ml)
r2 = 0.011, p<0.01
gestational week
B.
cortisol concentration (ng/ml)
r2 = 0.185, p<0.0001
gestational week
C.
17OH-progesterone (nM)
r2 = 0.036, p<0.0001
gestational week
D.
PFOS (ng/ml)
r2 = 0.052, p<0.0001
gestational week
E.
5cx-MEPP concentration (ng/ml)
r2 = 0.066, p<0.0001
gestational week
F.
7cx-MMeHP concentration (ng/ml)
r2 = 0.013, p<0.004
gestational week

Suppl Fig 2

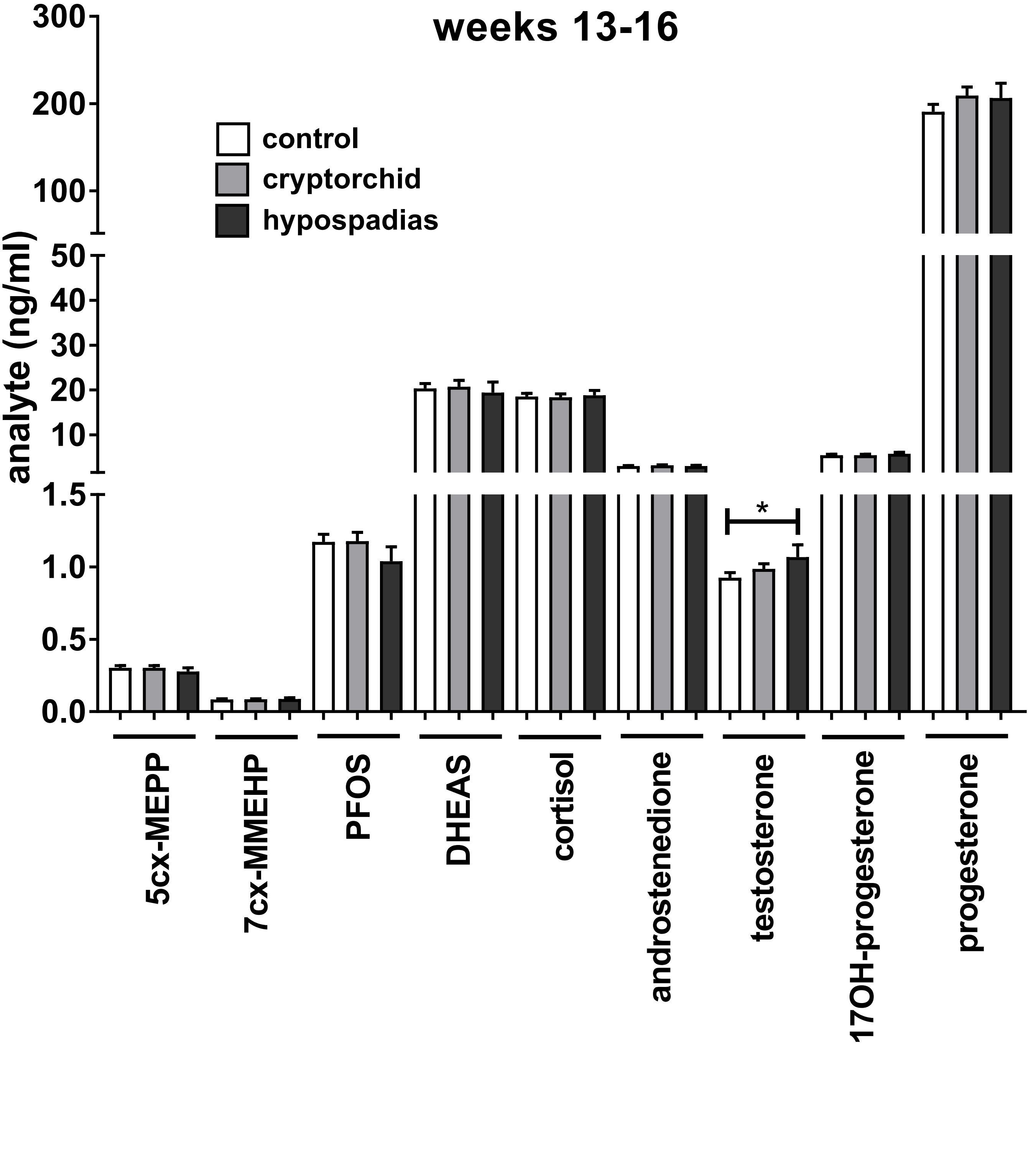
weeks 13-16
control
cryptorchid
hypospadias
analyte (ng/ml)
300
200
100
50
40
30
20
10
1.5
1.0
0.5
0.0
*
5cx-MEPP
7cx-MMEHP
PFOS
DHEAS
cortisol
androstenedione
testosterone
17OH-progesterone
progesterone

Suppl. Table 1 – Cases and controls combined (weeks 11-22; corrected for gestational week)

variables		INSL3	A4	T	DHEAS	17OH-P4	P4	cortisol	cotinine	PFOS	5cxMEPP	7cxMMEHP
**INSL3 MoM**	correlation	0.731	0.131	0.194	0.093	0.028	0.046	0.014	0.041	0.057	-0.072	-0.059
	significance	<0.001	0.005	<0.001	0.043	0.550	0.319	0.759	0.372	0.214	0.119	0.204
	df	470	470	470	470	470	470	470	470	470	470	470
**INSL3**	correlation		0.154	0.159	0.035	-0.039	0.025	-0.004	0.006	-0.056	-0.107	-0.104
	significance		0.001	0.001	0.445	0.402	0.582	0.938	0.894	0.226	0.020	0.023
	df		470	470	470	470	470	470	470	470	470	470
**A4**	correlation			0.481	0.305	0.459	0.512	0.458	0.113	0.191	0.051	-0.111
	significance			<0.001	<0.001	<0.001	<0.001	<0.001	0.014	<0.001	0.271	0.016
	df			470	470	470	470	470	470	470	470	470
**T**	correlation				0.359	0.398	0.386	0.381	0.039	0.240	0.144	-0.014
	significance				<0.001	<0.001	<0.001	<0.001	0.395	<0.001	0.002	0.755
	df				470	470	470	470	470	470	470	470
**DHEAS**	correlation					0.454	0.186	0.338	0.127	0.108	0.112	0.143
	significance					<0.001	<0.001	<0.001	0.006	0.019	0.015	0.002
	df					470	470	470	470	470	470	470
**17OH-P4**	correlation						0.392	0.506	0.250	0.243	0.189	0.001
	significance						<0.001	<0.001	<0.001	<0.001	<0.001	0.986
	df						470	470	470	470	470	470
**P4**	correlation							0.299	0.020	0.184	0.081	-0.037
	significance							<0.001	0.665	<0.001	0.080	0.425
	df							470	470	470	470	470
**cortisol**	correlation								125	0.297	0.118	0.045
	significance								0.006	<0.001	0.010	0.328
	df								470	470	470	470
**cotinine**	correlation									-0.033	0.006	-0.132
	significance									0.469	0.890	0.004
	df									470	470	470
**PFOS**	correlation										0.045	0.146
	significance										0.329	0.002
	df										470	470
**5cxMEPP**	correlation											0.174
	significance											<0.001
	df											470

NB. yellow indicates significant positive partial correlation (p<0.05); orange indicates significant negative partial correlation (p<0.05)

Suppl. Table 2 – Controls only (weeks 11-22; corrected for gestational week)

variables		INSL3	A4	T	DHEAS	17OH-P4	P4	cortisol	cotinine	PFOS	5cxMEPP	7cxMMEHP
**INSL3 MoM**	correlation	0.935	0.187	0.142	-0.014	-0.022	-0.066	0.015	0.087	-0.050	-0.111	-0.111
	significance	<0.001	0.007	0.040	0.837	0.751	0.343	0.827	0.207	0.468	0.109	0.108
	df	208	208	208	208	208	208	208	208	0.208	208	208
**INSL3**	correlation		0.202	0.177	-0.015	-0.041	-0.068	-0.001	0.064	-0.031	-0.064	-0.113
	significance		0.003	0.010	0.832	0.552	0.327	0.991	0.354	0.657	0.356	0.101
	df		208	208	208	208	208	208	208	208	208	208
**A4**	correlation			0.572	0.237	0.498	0.478	0.359	0.121	0.276	0.036	-0.191
	significance			<0.001	0.001	<0.001	<0.001	<0.001	0.081	<0.001	0.603	0.006
	df			208	208	208	208	208	208	208	208	208
**T**	correlation				0.188	0.414	0.455	0.343	0.045	0.290	0.090	-0.063
	significance				0.006	<0.001	<0.001	<0.001	0.520	<0.001	0.193	0.366
	df				208	208	208	208	208	208	208	208
**DHEAS**	correlation					0.322	0.159	0.113	0.059	0.181	0.039	0.065
	significance					<0.001	0.021	0.102	0.396	0.008	0.570	0.350
	df					208	208	208	208	208	208	208
**17OH-P4**	correlation						0.402	0.454	0.226	0.357	0.065	-0.038
	significance						<0.001	<0.001	0.001	<0.001	0.352	0.588
	df						208	208	208	208	208	208
**P4**	correlation							0.261	-0.020	0.273	0.028	-0.100
	significance							<0.001	0.777	<0.001	0.688	0.150
	df							208	208	208	208	208
**cortisol**	correlation								0.157	0.297	0.068	0.071
	significance								0.023	<0.001	0.329	0.309
	df								208	208	208	208
**cotinine**	correlation									-0.064	-0.038	-0.189
	significance									0.354	0.588	0.006
	df									208	208	208
**PFOS**	correlation										0.081	0.147
	significance										0.245	0.034
	df										208	208
**5cxMEPP**	correlation											0.226
	significance											0.001
	df											208

NB. yellow indicates significant positive partial correlation ($p<0.05$); orange indicates significant negative partial correlation ($p<0.05$)

Suppl. Table 3 – Cryptorchid only (weeks 11-22; corrected for gestational week)

variables		INSL3	A4	T	DHEAS	17OH-P4	P4	cortisol	cotinine	PFOS	5cxMEPP	7cxMMEHP
**INSL3 MoM**	correlation	0.658	0.118	0.248	0.118	0.040	0.124	0.013	0.024	0.119	-0.060	-0.042
	significance	<0.001	0.097	<0.001	0.099	0.573	0.081	0.857	0.739	0.095	0.399	0.556
	df	196	196	196	196	196	196	196	196	196	196	196
**INSL3**	correlation		0.199	0.178	0.090	0.003	0.136	0.012	-0.048	-0.098	-0.161	-0.112
	significance		0.005	0.012	0.207	0.961	0.056	0.866	0.505	0.170	0.023	0.116
	df		196	196	196	196	196	196	196	196	196	196
**A4**	correlation			0.549	0.431	0.526	0.584	0.544	0.135	0.129	0.073	-0.079
	significance			<0.001	<0.001	<0.001	<0.001	<0.001	0.058	0.070	0.304	0.270
	df			196	196	196	196	196	196	196	196	196
**T**	correlation				0.463	0.436	0.447	0.435	0.030	0.253	0.213	0.011
	significance				<0.001	<0.001	<0.001	<0.001	0.679	<0.001	0.003	0.881
	df				196	196	196	196	196	196	196	196
**DHEAS**	correlation					0.512	0.246	0.480	0.175	0.120	0.153	0.230
	significance					<0.001	<0.001	<0.001	0.014	0.093	0.031	0.001
	df					196	196	196	196	196	196	196
**17OH-P4**	correlation						0.456	0.577	0.288	0.169	0.332	0.332
	significance						<0.001	<0.001	<0.001	0.017	<0.001	<0.001
	df						196	196	196	196	196	196
**P4**	correlation							0.318	0.043	0.144	0.141	-0.017
	significance							<0.001	0.547	0.043	0.048	0.814
	df							196	196	196	196	196
**cortisol**	correlation								0.111	0.335	0.150	0.010
	significance								0.119	<0.001	0.034	0.888
	df								196	196	196	196
**cotinine**	correlation									0.017	0.079	-0.083
	significance									0.815	0.272	0.243
	df									196	196	196
**PFOS**	correlation										0.025	0.118
	significance										0.727	0.097
	df										196	196
**5cxMEPP**	correlation											0.119
	significance											0.096
	df											196

NB. yellow indicates significant <u>positive</u> partial correlation ($p<0.05$); orange indicates significant <u>negative</u> partial correlation ($p<0.05$)

Suppl. Table 4 – Hypospadias only (weeks 11-22; corrected for gestational week)

variables		INSL3	A4	T	DHEAS	17OH-P4	P4	cortisol	cotinine	PFOS	5cxMEPP	7cxMMEHP
**INSL3 MoM**	correlation	0.783	0.062	0.028	-0.062	-0.106	-0.146	-0.068	-0.020	0.043	-0.006	0.016
	significance	<0.001	0.630	0.829	0.635	0.414	0.256	0.598	0.875	0.742	0.966	0.903
	df	60	60	60	60	60	60	60	60	60	60	60
**INSL3**	correlation		0.003	0.056	-0.052	-0.220	-0.155	-0.040	0.022	0.072	-0.109	-0.016
	significance		0.982	0.663	0.691	0.086	0.228	0.756	0.867	0.576	0.400	0.899
	df		60	60	60	60	60	60	60	60	60	60
**A4**	correlation			0.132	0.020	0.221	0.360	0.406	0.058	0.130	0.089	-0.124
	significance			0.306	0.878	0.085	0.004	0.001	0.653	0.315	0.492	0.338
	df			60	60	60	60	60	60	60	60	60
**T**	correlation				0.288	0.207	0.012	0.239	0.066	0.015	0.210	-0.032
	significance				0.023	0.106	0.929	0.061	0.610	0.909	0.101	0.806
	df				60	60	60	60	60	60	60	60
**DHEAS**	correlation					0.465	0.003	0.214	0.125	-0.172	0.293	-0.076
	significance					<0.001	0.980	0.095	0.335	0.181	0.021	0.560
	df					60	60	60	60	60	60	60
**17OH-P4**	correlation						0.190	0.360	0.204	0.115	0.295	-0.031
	significance						0.138	0.004	0.111	0.374	0.020	0.811
	df						60	60	60	60	60	60
**P4**	correlation							0.287	0.077	0.096	0.129	-0.034
	significance							0.024	0.553	0.459	0.317	0.793
	df							60	60	60	60	60
**cortisol**	correlation								0.057	0.076	0.287	0.100
	significance								0.659	0.557	0.024	0.438
	df								60	60	60	60
**cotinine**	correlation									-0.108	-0.007	-0.096
	significance									0.402	0.956	0.459
	df									60	60	60
**PFOS**	correlation										-0.028	0.298
	significance										0.829	0.019
	df										60	60
**5cxMEPP**	correlation											0.237
	significance											0.064
	df											60

NB. yellow indicates significant positive partial correlation ($p<0.05$); orange indicates significant negative partial correlation ($p<0.05$)

Suppl. Table 5 – Cases and controls combined (weeks 13-16; corrected for gestational week)

variables		INSL3	A4	T	DHEAS	17OH-P4	P4	cortisol	cotinine	PFOS	5cxMEPP	7cxMMEHP
**INSL3 MoM**	correlation	0.960	0.136	0.156	0.034	-0.045	-0.015	0.009	0.039	-0.056	-0.113	-0.104
	significance	<0.001	0.011	0.003	0.529	0.407	0.785	0.861	0.472	0.299	0.036	0.052
	df	346	346	346	346	346	346	346	346	346	346	346
**INSL3**	correlation		0.138	0.147	0.028	-0.056	-0.022	-0.005	0.023	-0.068	-0.094	-0.109
	significance		0.010	0.006	0.606	0.298	0.676	0.931	0.672	0.203	0.079	0.041
	df		346	346	346	346	346	346	346	346	346	346
**A4**	correlation			0.479	0.278	0.438	0.478	0.455	0.091	0.187	0.038	-0.122
	significance			<0.001	<0.001	<0.001	<0.001	<0.001	0.089	<0.001	0.481	0.023
	df			346	346	346	346	346	346	346	346	346
**T**	correlation				0.273	0.397	0.334	0.360	-0.013	0.233	0.108	-0.043
	significance				<0.001	<0.001	<0.001	<0.001	0.804	<0.001	0.044	0.429
	df				346	346	346	346	346	346	346	346
**DHEAS**	correlation					0.372	0.121	0.237	0.069	0.083	0.132	0.116
	significance					<0.001	0.024	<0.001	0.198	0.121	0.014	0.031
	df					346	346	346	346	346	346	346
**17OH-P4**	correlation						0.357	0.524	0.221	0.270	0.207	-0.019
	significance						<0.001	<0.001	<0.001	<0.001	<0.001	0.718
	df						346	346	346	346	346	346
**P4**	correlation							0.283	0.009	0.216	0.085	-0.050
	significance							<0.001	0.873	<0.001	0.114	0.350
	df							346	346	346	346	346
**cortisol**	correlation								0.100	0.258	0.117	0.010
	significance								0.063	<0.001	0.030	0.846
	df								346	346	346	346
**cotinine**	correlation									-0.040	0.007	-0.112
	significance									0.452	0.897	0.036
	df									346	346	346
**PFOS**	correlation										-0.018	0.120
	significance										0.733	0.025
	df										346	346
**5cxMEPP**	correlation											0.133
	significance											0.013
	df											346

NB. yellow indicates significant <u>positive</u> partial correlation (p<0.05); orange indicates significant <u>negative</u> partial correlation (p<0.05)
